# Rapid Detection Assay for Infectious Bronchitis Virus Using Real-Time Reverse Transcription Recombinase-Aided Amplification

**DOI:** 10.3390/v17091172

**Published:** 2025-08-27

**Authors:** Nahed Yehia, Ahmed Abd El Wahed, Abdelsatar Arafa, Dalia Said, Ahmed Abd Elhalem Mohamed, Samah Eid, Mohamed Abdelhameed Shalaby, Rea Maja Kobialka, Uwe Truyen, Arianna Ceruti

**Affiliations:** 1 Reference Laboratory for Veterinary Quality Control on Poultry Production, Animal Health Research Institute, Agriculture Research Center, Giza 12618, Egypt; abd.arafa@gmail.com (A.A.); daliasaid@yahoo.com (D.S.); vet_t2050@yahoo.com (A.A.E.M.); samaheid@ymail.com (S.E.); 2 Institute of Animal Hygiene and Veterinary Public Health, Leipzig University, 04103 Leipzig, Germany; ahmed.abd_el_wahed@uni-leipzig.de (A.A.E.W.); rea_maja.kobialka@uni-leipzig.de (R.M.K.); truyen@vetmed.uni-leipzig.de (U.T.); 3 Department of Virology, Faculty of Veterinary Medicine, Cairo University, Cairo 12211, Egypt; mshalaby@cu.edu.eg

**Keywords:** infectious bronchitis virus, rapid diagnostic test, RT-RAA, recombinase-aided amplification

## Abstract

The infectious bronchitis virus (IBV) causes a severe infectious disease in poultry, leading to significant financial losses. The prevention and treatment of this disease are extremely challenging due to the virus’s rapid mutation. Therefore, quick diagnosis of IBV infections is crucial for controlling the disease. This study aimed to develop a real-time reverse transcription recombinase-aided amplification (RT-RAA) method for IBV. The most effective primer combination was selected for further validation. To determine the assay’s analytical sensitivity, a serial dilution from 10^5^ to 10^0^ EID_50_/mL was used, and the limit of detection was calculated. The assay could detect down to 10^2^ EID_50_/mL. The limit of detection (95% Confidence Interval) was 67 EID_50_ per reaction. There was no cross-reaction with common poultry diseases. When analyzing 39 clinical samples, RT-RAA and RT-PCR showed 100% diagnostic sensitivity and specificity. In conclusion, the IBV RT-RAA detection method is rapid, sensitive, and specific. This approach can be used to improve IBV diagnosis at the point of need.

## 1. Introduction

Infectious bronchitis (IB) is a highly contagious and economically important disease primarily affecting birds within the order Galliformes. The disease is caused by the infectious bronchitis virus (IBV), a positive-sense single-stranded RNA virus belonging to the genus Gammacoronavirus within the family Coronaviridae. Glycoprotein Spike (S), Nucleocapsid (N) protein, glycoprotein Membrane (M), and protein Envelope (E) make up the major structural proteins, which are important in genotyping classification of IBV [1].

IBV primarily targets the respiratory tract of the hosts but can also affect the kidneys, oviducts, and reproductive organs, leading to nephritis, reproductive disorders, poor weight gain, reduced egg production, and deterioration in egg quality [2]. The disease affects poultry of all ages and breeds. Outbreaks can spread rapidly through airborne transmission or contaminated equipment, feed, or personnel. The virus exhibits high genetic variability, resulting in the emergence of multiple serotypes and genotypes that often evade vaccine-induced immunity. This antigenic variation complicates control and prevention efforts, making timely and accurate diagnosis of circulating IBV strains an essential component of any control strategy [3].

Various poultry diseases, such as IBV, Newcastle disease (ND), infectious laryngotracheitis (ILT), and avian influenza (AI), display similar clinical symptoms, leading to frequent IB false diagnosis [4]. Therefore, laboratory diagnosis is essential to confirm an IBV outbreak. Many laboratory techniques, including molecular assays and serological assays, have been developed to identify IBV [5].

Traditional diagnostic methods for IBV include virus isolation in embryonated chicken eggs, immunofluorescence assays, and serological tests such as ELISA and hemagglutination inhibition. However, these methods are often labor-intensive, time-consuming, and may lack the sensitivity required for early detection, especially during the incubation period or in birds with low viral loads [3,6]. Major structural and non-structural proteins are encoded by the genome’s 5′-UTR-1a-1b-S-3a-3b-E-M-4b-4c-5a-5b-N-6b-UTRpoly (A) tail-3′ [7]. Molecular techniques such as reverse transcription-polymerase chain reaction (RT-PCR) are more sensitive and specific, and they have become the gold standard for IBV detection and genotyping [8]. However, these methods require expensive thermal cyclers, trained personnel, and access to laboratory infrastructure, which may not be available in remote or resource-limited areas. Furthermore, sample processing, nucleic acid extraction, and cold-chain requirements for reagents can delay the diagnostic process, limiting the practical utility of PCR-based methods in the field [9]. There is a need for rapid, accurate, cost-effective, and easily deployable IBV detection assays.

To overcome the limitations of conventional molecular diagnostics, isothermal amplification technologies have been developed, offering faster and more portable alternatives. Among these, recombinase-aided amplification (RAA) (formerly known as recombinase polymerase amplification) has gained significant attention for its simplicity, rapid reaction time, and minimal equipment requirements. RAA is an isothermal nucleic acid amplification technique that utilizes recombinase proteins to facilitate primer binding to the template DNA, along with strand-displacing DNA polymerase to amplify the target sequence without the need for thermal cycling. The reaction proceeds efficiently at low, constant temperatures (37 °C to 42 °C), enabling amplification in as little as 10 to 20 min [10]. When adapted for RNA viruses like IBV, RAA is combined with a reverse transcriptase enzyme in a single-step reaction known as reverse transcription recombinase-aided amplification (RT-RAA), which enables the direct amplification of viral RNA. This technology is particularly advantageous for rapid, on-site diagnostics and is well-suited to veterinary settings where timely decisions are crucial.

Several studies have validated the application of a real-time RT-RAA for the detection of several diseases, demonstrating excellent sensitivity, specificity, and speed [11]. Studies have successfully developed RT-RAA assays targeting H5 avian influenza, H9 avian influenza, and ILT [11,12,13]. The simplicity and rapid turnaround of RT-RAA make it especially useful during disease outbreaks, where immediate identification of the causative agent is essential for implementing control measures such as isolation, vaccination, or targeted biosecurity interventions.

In addition to its diagnostic efficiency, RT-RAA has practical advantages that allow it to be highly suitable for field use. The reagents can be lyophilized and stored at ambient temperatures, reducing dependence on the cold chain. The reaction can be performed using portable heaters or even simple heat blocks, and results can be interpreted visually using lateral flow strips without the need for laboratory-grade equipment. Portable fluorometers can be used for real-time detection. This ease of use is particularly beneficial in developing countries or rural areas with limited veterinary infrastructure [14]. Furthermore, RT-RAA is robust to many inhibitors commonly found in crude clinical or environmental samples, enabling direct testing from swabs, tissues, or feces with minimal processing [15]. Such features expand the usability of RT-RAA as a point-of-care diagnostic tool, promoting rapid surveillance and early detection, both of which are critical to controlling the spread of IBV in poultry populations.

The aim of this study was to develop a real-time RT-RAA test for rapid detection of IBV which can be deployed at point of need.

## 2. Material and Methods

### 2.1. Preparation of RNA Standard

The viral strain used for assay validation was based on the reference strain of Egyptian IBV viruses (IBV/Chicken/Egypt/RLQP/F148/2023 with GenBank accession number OR032917. The virus titer shown as EID_50_ (50% embryo infective dose)/mL was calculated using a previously described method [16].

### 2.2. RNA Extraction of Clinical Samples and Standard

Following the manufacturer’s instructions, the QIAamp Viral RNA Mini Kit was used to extract viral RNA from clinical samples and allantoic fluid (Qiagen, Hilden, Germany). The extraction process used two-hundred microliters from samples of tracheal swab suspensions on phosphate-buffered saline (PBS) or from allantoic fluid. After being eluted in a final volume of 50 µL, the RNA was kept at −80 °C until further use.

### 2.3. IBV RT-RAA Primers and Exo-Probe Design

To determine which combination produced the best RT-RAA assay sensitivity, two forward primers, three reverse primers, and one exo-probe were designed (Table 1). The conserved 5′-End UTR target region was selected after the alignment of 50 IBV complete genomes that represent most common genotypes of IBV (Supplementary Material Table S1). The MegAlign software 11.0.13 (DNA Star, Inc., Madison, WI, USA) was used to perform the analysis. The oligonucleotides were synthesized by Tib MolBiol (Berlin, Germany) and created in compliance with the Twist Amp exo RT kits handbook (Twist Dx, Cambridge, UK).

### 2.4. RT-RAA Reaction Conditions

The IBV RT-RAA was performed in a 50 µL reaction volume using fluorescent RT-RAA kits (QT Biotech Co., Ltd., Wuxi, China) according to the manufacturer’s instructions. In brief, the 50 µL reaction was performed according to the following formula: 2.1 µL of each RAA primers (10 pmol Conc.), 0.6 µL RAA exo-probe (10 pmol Conc.), 2.5 µL of magnesium acetate (14 mM Conc.), 25 µL of 4 × rehydration buffer, and 5 µL RNA template; then, PCR grade water was added to adjust the reaction volume up to 50 µL. This mix was added to the RAA strips containing a dried enzyme pellet. Heating and fluorescence measurements were conducted in an ESEQuant tube scanner (Qiagen, Hilden, Germany) at 42 °C for 20 min. The threshold time (TT) was determined by combining threshold and signals loop analysis confirmed by 1st derivative analysis in the tube scanner software.

### 2.5. Validation of IBV RT-RAA

#### 2.5.1. IBV RT-RAA Sensitivity

The analytical sensitivity of the RT-RAA assay was tested using a dilution range of titrated IBV from 10^5^ EID_50_/mL to 10^0^ EID_50_/mL in three replicates.

#### 2.5.2. IBV RT-RAA Specificity and Cross Reactivity

The specificity of the IBV RT-RAA assay was determined by testing six viruses causing respiratory symptoms: Highly Pathogenic Avian Influenza (H5N1, H7N1), Infectious Laryngotracheitis Virus (ILTV), Newcastle Disease Virus, and *Mycoplasma gallisepticum*.

#### 2.5.3. Clinical Performance of IBV RT-RAA

The clinical performance of the IBV RT-RAA assay was evaluated in comparison to real-time RT-PCR using 39 tracheal swabs obtained from field cases in Egypt from routine surveillance. The real-time RT-PCR was performed with the Quantitect probe RT-PCR kit (Qiagen, Valencia, CA, USA) and using previously described oligonucleotides [17]. A total of 24 samples were positive, with Ct values from 14 to 35, and 15 samples were negative.

### 2.6. Statistical Analysis

The tube scanner software (Qiagen, Hilden, Germany) was used to calculate the RT-RAA TT by using the first derivatives of the real-time fluorescence signal measurement. The cut-off value was the value of the negative control. The RT-RAA’s clinical sensitivity and specificity were assessed using the previously published standard formula [18]. PRISM (GraphPad Software Inc., version 6.0 Mac, San Diego, CA, USA) was used for linear regression analysis of TT values and dilution ranges. The probit analysis for limit of detection determination was calculated using RStudio (2024.04.0).

The Pearson correlation coefficient r between real-time RT-PCR CT values and IBV RT-RAA TT was calculated using linear regression analysis and visualized using PRISM (Graphpad Software Inc. version 6.0 Mac, San Diego, CA, USA) [19].

## 3. Results

### 3.1. Selection of the Primer Sets

**Figure 1:**
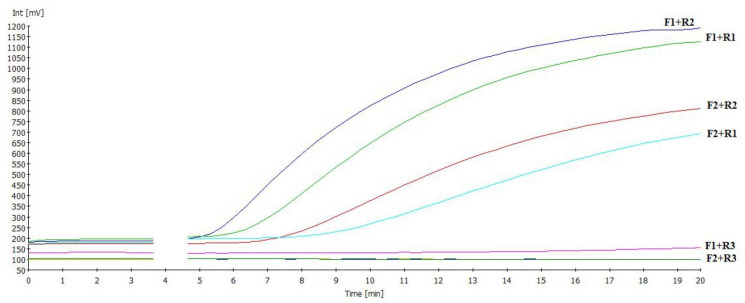
Different primer combinations were tested using 10^5^ EID_50_/mL of IBV standard to determine the best combination.

The amplification of 174–191 bp of 10^5^ EID_50_/mL per RT-RAA reaction mixture was tested for all primer/probe combinations that target the 5-end untranslated region of IBV (Table 1). Out of all the primer combinations, the primer pair RAA-F1 + IBV RAA-R2 produced the best amplification of 10^5^ EID_50_/mL, with signals appearing at 5.5 min (Figure 1, in bold). As a result, the RT-RAA assay was further validated using this primer set.

### 3.2. Analytical Sensitivity of IBV RT-RAA Assay

**Figure 2:**
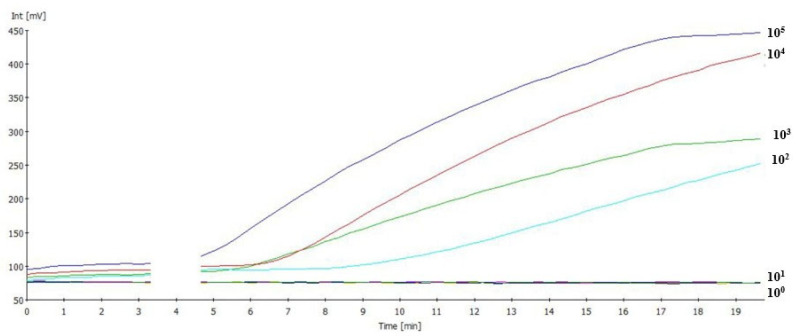
Analytical sensitivity of IBV RAA-F1 + IBV RAA-R2 by using dilution ranging from 10^5^ EID_50_/mL to 10^0^ EID_50_/mL. Dilution 10^2^ EID_50_/mL was detected.

**Figure 3:**
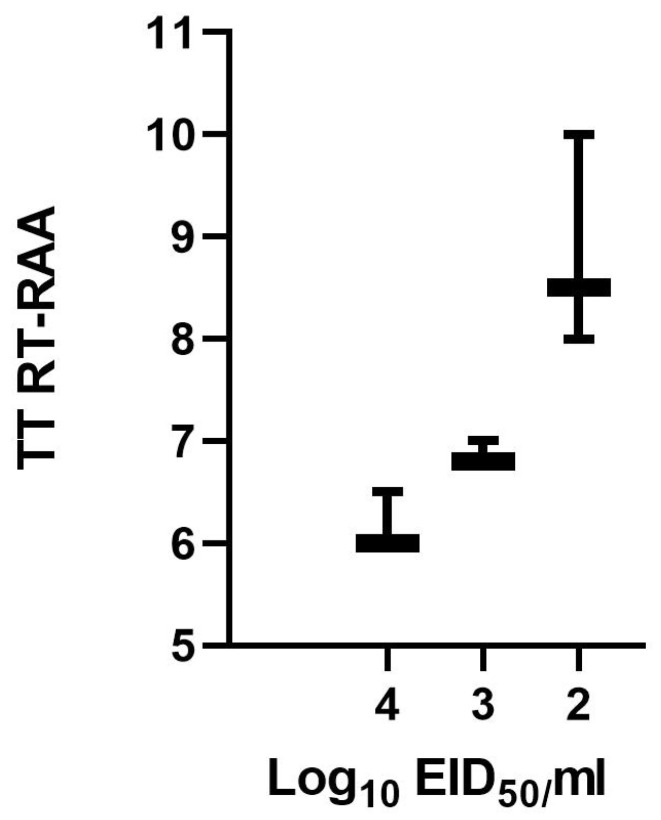
Semi-logarithmic regression of data collected from three IBV RT-RAA test runs using PRISM Software. Results were obtained in 5–10 min. The assay was able to detect down to 10^2^ EID_50_/mL in all runs. The thick horizontal line represents the mean value.

To determine the analytical sensitivity of IBV RT-RAA assay, a dilution range of 10^5^–10^0^ EID_50_/mL of the IBV RNA standard was used in three replicates (Figure 2). The previously chosen primer combination detected all dilution replicates down to 10^2^ EID_50_/mL in 8–10 min. The dilutions 10^1^ to 10^0^ EID_50_/mL were all negative. A semi-logarithmic regression of the data collected from three IBV RT-RAA test runs on the RNA standard using PRISM yielded results between 5–10 min (Figure 3). The limit of detection (95% CI) was approximately 67 EID_50_/reaction.

### 3.3. Analytical Specificity of IBV RT-RAA Assay

**Figure 4:**
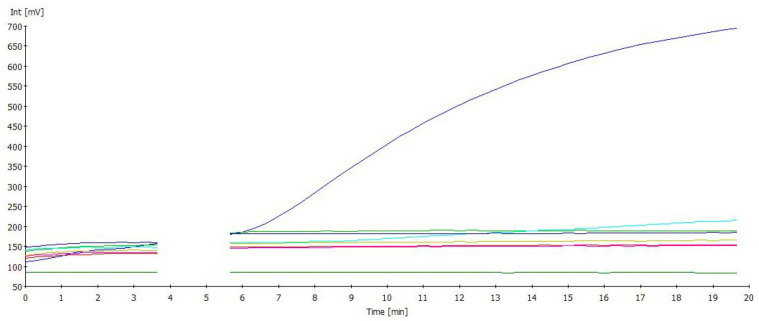
IBV RT-RAA specificity test showed no amplification curves for H5N1, H7N1, ILT, NDV, and *Mycoplasma gallisepticum.* Only IBV was detected.

The IBV RT-RAA assay was shown to be specific when tested with various pathogens that produce respiratory symptoms in poultry, such as H5N1, H7N1, ILT, NDV, and *Mycoplasma gallisepticum*. Only IBV produced amplification (Figure 4).

### 3.4. Clinical Performance of IBV RT-RAA Assay

**Figure 5:**
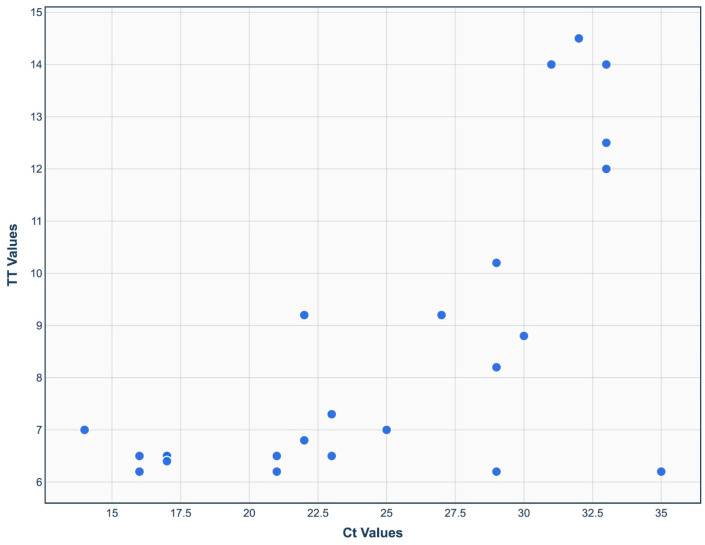
Comparison between TT value of real-time RT-RAA and Ct value of real-time RT-PCR. A moderate correlation was found (r^2^ = 0.673).

The RT-RAA assay was compared to real-time RT-PCR assay by evaluating it with thirty-nine clinical samples. A sensitivity and specificity of 100% was determined. The positive clinical samples were all detected with the IBV RT-RAA with TT values between 6.2–14 min. A moderate correlation between the Ct of the real-time PCR and the TT of the RT-RAA was found (Figure 5). The fifteen RT-PCR negative samples were also determined as negative by IBV RT-RAA.

## 4. Discussion

IBV can cause significant financial losses in the poultry industry due to immunosuppression, weight loss, decreased egg production, malformed eggs, and costs related to immunization and treatment [20,21]. Rapid and precise diagnosis, along with effective control strategies, are essential for managing IBV in poultry. Therefore, this study aimed to develop a simple, field-ready, real-time RT-RAA assay for IBV detection. The assay could detect as low as 10^2^ EID_50_/mL and achieved 100% clinical sensitivity and specificity.

Since IB’s clinical symptoms and pathological alterations resemble those of ND and ILT, it is frequently misdiagnosed. Although real-time RT-PCR and is conventional viral isolation are sensitive and specific methods for diagnosing the virus, they still have drawbacks, such as the need for highly trained personnel and the requirement for costly and heavy equipment [22].

A rapid, accurate, sensitive, and deployable method for isothermal amplification is reverse transcription recombinase-assisted amplification [15]. Because of its simplicity, rapid turnaround time, and lack of high temperature cycling, the entire reaction is ideal for usage in non-laboratory testing settings [10].

In the RAA assay, primer design remains a major bottleneck because, aside from a few guidelines, regulations are not explained and automated software design is still not feasible. The primers of the devised test were set in a highly conserved region of the viral genome, similar to the real-time RT-PCR [23]. In this study, the UTR gene was used to develop the RT-RAA technique and was validated with local clinical samples. Here, universal primers that are present in all serotypes of infectious bronchitis can bind to the UTR region. UTRs are generally considered more conserved than M genes, due to their regulatory functions [24]. For sensitive amplification of the IBV viral RNA standard, six potential combinations were examined. Only one primer set detected 10^2^ EID_50_/mL in 8–10 min. The IBV RT-RAA is highly specific and cannot identify distinct pathogens causing comparable respiratory symptoms in chickens or other seemingly healthy birds.

The IBV RT-RAA detected all real-time RT-PCR positive clinical samples. The remarkable sensitivity of the proposed assay is demonstrated by the fact that many samples with high Ct values of about 35 in real-time RT-PCR were recognized as positive by RT-RAA in less than 7 min. Since the RAA reaction is explosive and outruns the PCR reaction’s repetitive, regular amplification cycles, there was only a moderate correlation between the Ct values of the real-time RT-PCR and the TT values of the RT-RAA. Diverse correlations have been described for different RAA assays [25].

Simple handheld heating devices could be used to carry out RAA operations rather than thermal cyclers, which are required for conventional and real-time PCR. Rapid on-site diagnosis is more appropriate because RAA reagents are cold-chain independent [26,27]. Various assays for the molecular detection of IBV and NDV using reverse transcription loop-mediated isothermal amplification (RT-LAMP) were developed [28,29]. Unlike RAA, LAMP needs six primers, which hinders sensitivity for viruses with significant mutation rates, such as IBV and NDV. Also, RAA-lateral flow is used for the identification of infectious bronchitis [13,30]. However, due to its lower sensitivity, dependence on visualization, requirement for antibody production, and inaccurate sample volume, there is the probability of producing false positive results [31,32]. Although real-time fluorescence-based RAA offers significant advantages, further research in this field could lead to the development of quantification methods, differentiation between vaccine-related and field strains, and multiplex detection formats. Additionally, further clinical testing coupled with rapid extraction methods would underscore the potential of the developed assay at point of need.

In conclusion, recombinase-aided amplification provides a rapid, accurate, targeted, and field-deployable substitute for conventional IBV diagnostic techniques. Due to its isothermal properties and its capacity to identify viral RNA in a matter of minutes, it has the potential to revolutionize veterinary diagnostics. The poultry industry is continuously threatened by IBV variants. Implementing RT-RAA can improve IBV outbreak response diagnosing the virus in the field. This can lead to optimized vaccination regimens and increase the sustainability and productivity of poultry farming.

## Figures and Tables

**Table 1:** Primers and exo-probe used for IBV RT-RAA assay development.

Name	Sequence	Nucleotide Positions
**IB-Exo-probe**	5‘CAACCCCTGAGGTGACAGGTTCTGGTGG(BHQ1-dT)(THF)(FAM-dt)TTAGTGAGCAGACA-PH3‘	467–513
**I.B-F1**	**5‘TCACCTCCCCCCACATACCTCTAAGGGCTTTT3‘**	**389–420**
**I.B-F2**	5‘TCCCCCCACATACCTCTAAGGGCTTTTGAG3‘	368–399
**I.B-R1**	5‘TTAGGCTTGAAGCCATGTTGTCACTGTCTA3‘	518–547
**I.B-R2**	**5‘ATACTCCCTGTTTTAGGCTTGAAGCCATGTT3‘**	**529–559**
**I.B-R3**	5‘TTTAGGCTTGAAGCCATGTTGTCACTGTCTAT3‘	517–548

G = guanin, A = adenine, T = thymine, C = cytosine, THF = tetrahydrofuran, BHQ = Black Hole Quencher, FAM = fluorescein amidite.

## Data Availability

The raw data supporting the conclusions of this article will be made available by the authors on request.

## Supplementary Materials

The following supporting information can be downloaded at: https://www.mdpi.com/article/10.3390/v17091172/s1, Table S1: Avian infectious bronchitis virus strains used for alignment and primer sequence determination.
