# Correction: Yehia et al. Rapid Detection Assay for Infectious Bronchitis Virus Using Real-Time Reverse Transcription Recombinase-Aided Amplification. *Viruses* 2025, *17*, 1172

**DOI:** 10.3390/v18010098

**Published:** 2026-01-12

**Authors:** Nahed Yehia, Ahmed Abd El Wahed, Abdelsatar Arafa, Dalia Said, Ahmed Abd Elhalem Mohamed, Samah Eid, Mohamed Abdelhameed Shalaby, Rea Maja Kobialka, Uwe Truyen, Arianna Ceruti

**Affiliations:** 1 Reference Laboratory for Veterinary Quality Control on Poultry Production, Animal Health Research Institute, Agriculture Research Center, Giza 12618, Egypt; 2 Institute of Animal Hygiene and Veterinary Public Health, Leipzig University, 04103 Leipzig, Germany; 3 Department of Virology, Faculty of Veterinary Medicine, Cairo University, Cairo 12211, Egypt

## Error in Figure 5

In the original publication [1], there was a mistake in Figure 5 as published. The x-axis displays erroneously formatted values. The corrected Figure 5 appears below. The authors state that the scientific conclusions are unaffected. This correction was approved by the Academic Editor. The original publication has also been updated.

## Figures and Tables

**Figure 5:**
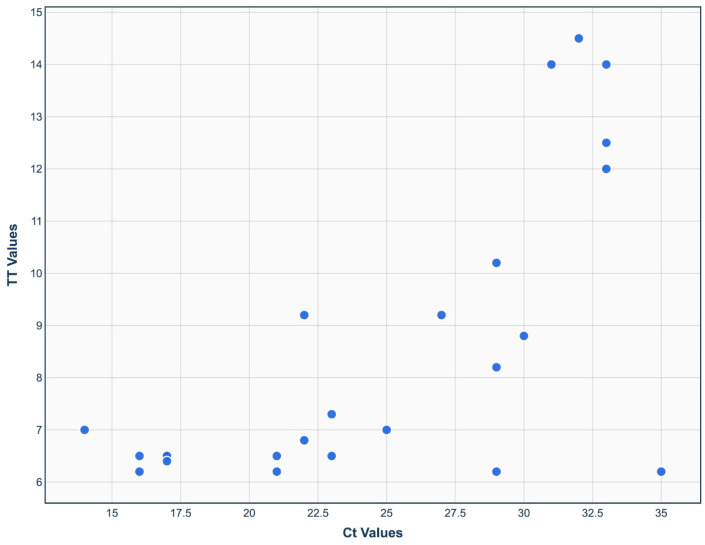
Comparison between TT value of real-time RT-RAA and Ct value of real-time RT-PCR. A moderate correlation was found (r^2^ = 0.673).
