# Supplementary material for: Rapid Detection Assay for Infectious Bronchitis Virus Using Real-Time Reverse Transcription Recombinase-Aided Amplification
Source: Viruses. 2025 Aug 27;17(9):1172. doi: 10.3390/v17091172 (PMC12474456; doi:10.3390/v17091172)
Supplement: Supplementary file 1 [file viruses-17-01172-s001.zip › viruses-3769003-supplementary.pdf]

Supplementary table S1. Avian infectious bronchitis virus strains used for alignment and primer sequence determination.

Strain	Accession number
Avian infectious bronchitis virus strain Mass 41	AY851295
Avian infectious bronchitis virus isolate IBV-p65	DQ001339
Avian infectious bronchitis virus isolate IBV-EP3	DQ001338
Avian infectious bronchitis virus isolate Peafowl/GD/KQ6/2003	AY641576
Avian infectious bronchitis virus isolate BJ	AY319651
Avian infectious bronchitis virus isolate SAIBK	DQ288927
Avian infectious bronchitis virus partridge/GD/S14/2003	AY646283
Infectious bronchitis virus isolate B17	KT203557
Infectious bronchitis virus isolate TW2575/98	DQ646405
Infectious bronchitis virus serotype California 99	AY514485
Infectious bronchitis virus strain A2	EU526388
Infectious bronchitis virus serotype JMK	GU393338
Infectious bronchitis virus serotype Iowa 97	GU393337
Infectious bronchitis virus serotype Holte	GU393336
Infectious bronchitis virus serotype H120	GU393335
Infectious bronchitis virus serotype Gray	GU393334
Infectious bronchitis virus serotype FL18288	GU393333
Infectious bronchitis virus serotype Delaware 072	GU393332
Infectious bronchitis virus serotype Cal56b	GU393331
Infectious bronchitis virus strain M41	DQ834384
Infectious bronchitis virus strain H52	EU817497
Infectious bronchitis virus strain CK/CH/LSD/05I	EU637854
Infectious bronchitis virus strain Sczy3	JF732903
Infectious bronchitis virus strain ArkDPI11	EU418976
Infectious bronchitis virus strain ck/CH/LDL/101212	JF828981
Infectious bronchitis virus strain ck/CH/LHLJ/100902	JF828980
Infectious bronchitis virus isolate GX-YL9	HQ850618
Infectious bronchitis virus isolate GX-YL5	HQ848267
Infectious bronchitis virus isolate CK/CH/XDC- 2/2013	KM213963
Infectious bronchitis virus strain ck/CH/LZJ/111113	JX195176
Infectious bronchitis virus strain ck/CH/LDL/091022	JX195175
Infectious bronchitis virus strain Ck/CH/LGD/120724	KC119407
Infectious bronchitis virus strain Ck/CH/LGD/120723	KC013541
Infectious bronchitis virus strain ck/CH/LJL/111054	KC506155
Infectious bronchitis virus isolate IBVUkr27-11	KJ135013
Infectious bronchitis virus strain CK/CH/LGX/091109	KF411041
Infectious bronchitis virus strain CK/CH/LLN/111169	KF411040
Infectious bronchitis virus isolate KM91	JQ977698
Infectious bronchitis virus isolate SNU8067	JQ977697
Infectious bronchitis virus isolate YN	JF893452
Infectious bronchitis virus strain ck/CH/LDL/97I substrain P115	JX195178
Infectious bronchitis virus strain ck/CH/LDL/97I substrain P5	JX195177
Infectious bronchitis virus strain Arkansas Vaccine	GQ504721
Infectious bronchitis virus strain Mass41 Vaccine	GQ504725
Infectious bronchitis virus strain Massachusetts	GQ504724
Infectious bronchitis virus strain Georgia 1998 Vaccine	GQ504723
Infectious bronchitis virus strain Georgia 1998 pass8	GQ504722
Infectious bronchitis virus strain Arkansas DPI	GQ504720
Infectious bronchitis virus strain Mass41 1985	FJ904723
Infectious bronchitis virus strain Mass41 1979	FJ904722
